# Early symptoms of autism spectrum disorder (ASD) in 1–8 year old children with sex chromosome trisomies (XXX, XXY, XYY), and the predictive value of joint attention

**DOI:** 10.1007/s00787-022-02070-y

**Published:** 2022-09-15

**Authors:** Nienke Bouw, Hanna Swaab, Nicole Tartaglia, Rebecca L. Wilson, Kim Van der velde, Sophie van Rijn

**Affiliations:** 1 Clinical Neurodevelopmental Sciences, Faculty of Social and Behavioral Sciences, Leiden University, PO Box 9500, 2300 RA Leiden, The Netherlands; 2 Leiden Institute for Brain and Cognition, Leiden, The Netherlands; 3 Developmental Pediatrics, University of Colorado School of Medicine, Children’s Hospital Colorado, Aurora, CO USA; 4 Department of Pediatrics, University of Colorado School of Medicine, Aurora, CO USA; 5 Department of Paediatric Neurology, UZ Brussel, Jette, Belgium

**Keywords:** Sex chromosome trisomy, Autism spectrum disorder, Joint attention, Klinefelter syndrome, Trisomy X

## Abstract

The objective of the present study is to investigate the impact of Sex Chromosome Trisomy (SCT; XXX, XXY, XYY) on the early appearance of Autism Spectrum Disorder (ASD) symptoms, and the predictive value of Joint Attention for symptoms of ASD. SCTs are specific genetic conditions that may serve as naturalistic ‘at risk’ models of neurodevelopment, as they are associated with increased risk for neurobehavioral vulnerabilities. A group of 82 children with SCT (aged 1–8 years) was included at baseline of this longitudinal study. Joint Attention was measured at baseline with structured behavior observations according to the Early Social Communication Scales. ASD symptoms were assessed with the Modified Checklist for Autism in Toddlers questionnaire and Autism Diagnostic Interview-Revised in a 1-year follow-up. Recruitment and assessment took place in the Netherlands and in the United States. The results demonstrate that ASD symptoms were substantially higher in children with SCT compared to the general population, with 22% of our cohort at clinical risk for ASD, especially in the domain of social interaction and communication. Second, a predictive value of Joint Attention was found for ASD symptoms at 1-year follow-up. In this cohort, no differences were found between karyotype-subtypes. In conclusion, from a very early age, SCT can be associated with an increased risk for vulnerabilities in adaptive social functioning. These findings show a neurodevelopmental impact of the extra X or Y chromosome on social adaptive development associated with risk for ASD already from early childhood onward. These findings advocate for close monitoring and early (preventive) support, aimed to optimize social development of young children with SCT.

## Introduction

Sex Chromosome Trisomies (SCTs) are among the most common chromosomal aneuploidies in humans [1], with a prevalence of 1:650–1:1000 of live births [2]. SCT, the presence of an extra X or Y chromosome, leads to the chromosomal patterns of 47,XXX in girls (Triple/Trisomy X), and 47,XXY (Klinefelter syndrome) and 47,XYY (XYY syndrome) in boys. SCT is characterized by a mild physical phenotype shared across SCT variants, with minimal atypical facial characteristics, tall stature, and low muscle tone [3]. On social behavioral level, SCT is associated with increased risk for challenges in social adaptive functioning, including shyness, social immaturity, difficulties in forming interpersonal relationships, increased levels of social anxiety, social impulsivity, and impairments in underlying social-cognitive mechanisms (see for reviews: [4–7]).

The severity of social behavioral vulnerabilities in school-aged children, adolescents, and adults with SCT is illustrated by an increased level of symptoms and clinical diagnoses of Autism Spectrum Disorder (ASD; see for a review: [8]). Previous studies determining the impact of SCT on ASD symptomatology have focused on populations with broad age ranges, including participants from middle childhood to adulthood. On average across these studies, depending on the ascertainment methods, diagnostic measurements and criteria used, about 15% (range 10.8–20%) of individuals with 47,XXX; 18% (range 10–27%) of individuals with 47,XXY and 30% (range 19–43%) of individuals with 47,XYY meet the full criteria of a clinical diagnosis of ASD [5, 9, 10]. Thus, compared to a worldwide prevalence rate of ASD of 0.6% in the general population [11], the prevalence of ASD has been shown to be seriously higher in SCT. Even when the most conservative prevalence rate is considered and acknowledging some level of ascertainment bias in previous studies, there is consistent evidence that SCT is associated with a significantly elevated risk for ASD relative to population estimates. However, information on early developmental pathways in young children with SCT before the age of 6 years precursing these social impairments later in life is extremely limited, as shown by a review [7]. This is unfortunate, as early childhood is a period in which brain networks that biologically underpin social (cognitive) development rapidly mature and specialize [12]. Consequently, early childhood serves as a key period to acquire social emotional and communicative developmental milestones [13]. Therefore, the current study aims to investigate the early appearance of ASD symptoms in young children with SCT.

Relevant to this developmental perspective is the notion that the presence of an additional X or Y chromosome is known to convergently impact the maturation of brain functions and networks involved in social adaptive cognitive and behavioral development, often referred to as the ‘social brain’ [14, 15]. Atypical brain maturation may be expressed in an impaired development of social-cognitive functions, necessary to shape social and communicative behavior in everyday life [16]. From this bottom–up neuropsychological perspective, having an extra X or Y chromosome may compromise social development and contribute to symptoms and clinical diagnoses of neurodevelopmental disorders such as ASD. Interestingly, since SCT can be identified as early as prenatally in contrast to ASD, early social developmental pathways can be studied prospectively. Identifying risk for ASD symptoms in genetic conditions, such as SCT, may therefore give insights of etiological pathways leading to complex behavioral phenotypes, thereby serving as a naturalistic ‘at risk’ model of neurobehavioral development [17]. This investigation therefore explores the question how early social developmental pathways in young children with SCT may lead to social impairment later in life, as expressed in symptoms of ASD.

A pivotal dimension of infant social cognition that serves as an important milestone in early typical social development and as an early precursor for ASD is Joint Attention [18–20]. Joint Attention refers to the active capacity to coordinate attention between interactive social partners to share an awareness of an objects or event in their environment [21]. In typical development, Joint Attention begins to emerge in the first 6 months of life and continues to develop at least until the age of 3 years old [22]. The early development of Joint Attention is manifest as two behavioral patterns: (1) responding to Joint Attention, referring to the ability to follow the direction of gaze and gestures of others toward the object or event in their environment (also referred to as ‘gaze following’), and (2) initiating Joint Attention, referring to the spontaneous use of direction of gaze and gestures to share or direct the attention of others toward the object or event, important for social understanding [23]. In typical early childhood, Joint Attention is an important social-cognitive mechanism exposing the child to social experiences of perspective sharing with others. Observations of Joint Attention provide important information about the development of mental processes that are crucial for subsequent components of social and cognitive development that impact the understanding of and responding to social relevant information. For example, differences in Joint Attention abilities among young children are associated with language and global cognitive development (e.g., [24, 25], and with social adaptation and self-regulation in preschool and school-aged children (e.g., [26, 27]). Accordingly, Joint Attention difficulties serve as a specific ‘red flag’ during social development of young children who are on developmental trajectories toward a clinical diagnosis of ASD, besides other pathologies like intellectual disabilities or specific language impairments [28]. Also in SCT, the development of Joint Attention abilities has proven to be vulnerable, as difficulties were found in the accuracy to spontaneously follow point and gaze gestures in children with SCT between 1 and 7 years old [29]. Joint Attention can be reliably and objectively measured in behavioral observations and are feasible to implement as a marker for ‘at-risk’ subsequent developmental screening of social development [30]. Because of the well-supported developmental continuity between early Joint Attention and social (cognitive) and communicative development, the present study aims to explore whether Joint Attention abilities in young children with SCT longitudinally predict ASD symptoms in SCT.

Taken together, the primary aim of the current study is to investigate early pathways to social impairment as expressed in ASD symptoms in an international sample of young children with SCT, aged 1–8 years. In addition to this main question, the predictive value of early Joint Attention on ASD symptoms 1 year later is explored to understand how early social-cognitive mechanisms may predict ASD symptoms in SCT. By identifying the effect of specific karyotype-subtype and possible recruitment bias, this study allows for an investigation of phenotypic differences within the SCT group. Based on the relevance of the extra X and Y chromosome on development of brain networks underlying social functioning and reported vulnerability for social adaptive functioning in individuals with SCT, we hypothesize that on average, children with SCT may show increased levels of ASD symptoms, and that Joint Attention difficulties predict these symptoms over time.

Due to advances in noninvasive prenatal testing technology, the number of prenatal diagnoses of SCT is growing [3]. Given this rise in prenatal diagnoses of SCT, there is not only the opportunity to prospectively investigate early social development, but also the great need to gather knowledge on the early development of children with SCT. These studies will help to identify early targets for monitoring and early intervention, leading to improved clinical care and developmental outcomes for young children with SCT.

## Methods

### Recruitment

The present study is part of a larger ongoing longitudinal study (the TRIXY Early Childhood Study—Leiden, The Netherlands), which includes children with SCT and nonclinical controls aged 1–8 years. The TRIXY Early Childhood Study aims to identify neurodevelopmental risk in young children with an extra X or Y chromosome.

Children with SCT were recruited and assessed at two sites: the Trisomy of the X and Y chromosomes (TRIXY) Expert Center at Leiden University (LUBEC) in Leiden, The Netherlands, and the eXtraordinary Kids Clinic in Developmental Pediatrics at Children’s Hospital Colorado in the USA. Children in the SCT group were recruited in cooperation with the clinical genetics and developmental pediatrics departments (from The Netherlands and Colorado, USA), as well as through patient-advocacy groups and social media postings. Recruitment strategy was assessed, and three subgroups were identified: (1) ‘active prospective follow-up’, which included families who were actively followed after prenatal diagnosis (52.4% of the SCT group), (2) ‘Information seeking parents’, which included families who were actively looking for more information about SCT without having specific concerns about the behavior of their child either after a prenatal or postnatal diagnosis (26.8% of the SCT group), and (3) ‘Clinically referred cases’, which included families seeking professional help based on specific concerns about their child’s development either after a prenatal or postnatal diagnosis (20.7% of the SCT group). All participants (child and parents) were Dutch or English speaking, and all children had normal or corrected-to-normal vision and did not have a history of traumatic brain injury or seizure disorder.

### Participants

We obtained written informed consent from the parents/guardians of all participating children, according to the Declaration of Helsinki. This study was approved by the Ethical Committee of Leiden University Medical Center, The Netherlands, and the Colorado Multiple Institutional Review Board (COMIRB) in Colorado, USA.

A group of 82 children with SCT (range 1–7 years old; *M*_age_ = 3.61, *SD* = 1.90) was included at baseline of the study. The study sample consisted of 24 girls with 47,XXX (29.2%), 41 boys with 47,XXY (50.0%), and 17 boys with 47,XYY (20.7%). The diagnosis of SCT was defined by trisomy in at least 80% of the cells, which was confirmed by standard karyotyping. Fifty-six children (68.3%) were diagnosed prenatally and 26 children were diagnosed postnatally (31.7%). Nineteen out of 41 boys with 47,XXY had received testosterone treatment (46.3%).

Parental education of caregivers was assessed at baseline, according to the criteria of Hollingshead [31]. Scores of this scale include: 0 (no formal education), 1 (less than seventh grade), 2 (junior high school), 3 (partial high school), 4 (high school graduate), 5 (partial college or specialized training), 6 (standard college/university graduation), and 7 (graduate/professional training). If two parents were available, level of education was averaged over both parents. Mean parental education was 5.95 (*SD* = 0.93). 95.6% of all parents indicated that their child has a second caregiver.

### Measurements and instruments

#### Joint attention

Joint Attention abilities were measured at baseline in a systematic behavior observation of a structured 20-min play situation, the Early Social Communication Scales [ESCS; 32]. The ESCS is designed to measure the development of different dimensions of early non-verbal communication. Children were presented with a series of wind-up toys, hand-operated toys, and a book to look at with the experimenter that have been designed to elicit social and communicative bids with the experimenter. In addition, the experimenter presented the child with two sets of gaze-following trials. Data from the ESCS domains of Initiating Joint Attention and Responding Joint Attention were examined in this study. Following the procedures described by [32], the ratings of Joint Attention during fixed time intervals were scored by trained independent raters. Coding consists of noting the frequency of occurrence of Joint Attention. Raters were not involved in the assessment, and blind to the child’s karyotype. See Table 1 for a description of initiations of Joint Attention and responses to Joint Attention. The Joint Attention data for 1–2 year old children with SCT were already published in [29].

**Table 1:** Description of early joint attention behavior coded during social interactions (based on [32])

Joint attention		
Initiating joint attention	Eye contact	Child makes eye contact with examiner while manipulating or touching an inactive mechanical toy
	Alternate	Child alternates looking at active mechanical toy or a toy in their hand and the examiner’s eyes
	Point	Child extends index finger toward toy within reach or to part of the room (e.g., posters)
	Show	Child extends toy toward the examiner’s face
Responding to joint attention	Look	Child turns head and eyes in the direction of the examiner’s pointing gesture, or to the appropriate area of a book

#### Symptoms of ASD in 2–4 year old children: M-CHAT

The Modified Checklist for Autism in Toddlers (M-CHAT) was administrated at follow-up to explore early signs of ASD in 2–4 year old children with SCT. The M-CHAT is an Autism Spectrum Disorder screener with 20 questions about current skills and behaviors, using a yes/no format [33]. Examples of items are: ‘Does your child play pretend or make-believe?’ and ‘Does your child respond when you call his or her name?’ The Dutch version was translated from the original English version using a ‘forward-back’ translation approach by a bilingual native speaker of both languages [34, 35]. The M-CHAT was administrated to detect ASD symptoms in children with SCT aged 2–4 year children. Presence of non-typical behavior was assigned a score of 1 and total score was interpreted. According to [33], total score of 0–2 suggests a negative low risk for ASD, total score of 3–7 as positive medium risk for ASD, and total score of 8–20 as positive high risk for ASD. The M-CHAT has shown to have adequate sensitivity and specificity [33].

#### Symptoms of ASD in 4–8 year old children: ADI-R

The Autism Development Interview-Revised (ADI-R) is a structured parent-report interview and recognized as the golden standard for establishing a clinical diagnosis of ASD [36, 37], and was administrated at follow-up to investigate ASD symptoms in 4–8 year old children with SCT. The ADI-R is based on DSM-IV and ICD-10 diagnostic criteria for ASD, which includes over 100 items focused and generates algorithm scores for each of three subdomains of ASD symptomatology: (a) qualitative impairments in reciprocal social behavior (Social Interaction), (b) deficits in communication and language development (Communication), and (c) restricted range of interest and/or stereotypic behaviors (Restricted and Repetitive Behavior/Interests). The ADI-R interviews were scored by trained independent raters on the following scale: 0: “behavior of the type specified in the coding is not present”; 1: “behavior of the type specified is present in an abnormal form, but not sufficiently severe or frequent to meet the criteria for a 2″; 2: “definite abnormal behavior”; 3: “extreme severity of the specified behavior”; 7 for “definite abnormality in the general area of the coding, but not of the type specified”; 8 for “not applicable”; and 9 for “not known or not asked”. To be consistent with DSM-5 diagnostic criteria for ASD [38], items from the Social and Communication domains were considered together. A sum of scores on the Social Interaction + Communication domain and on the Restricted and Repetitive Behaviors/Interests domain resulted in an ADI-R total score. We used the diagnostic algorithm, which is based on the (retrospective) functioning at age 4–5 years. For each primary domain of ASD, a cut-off score is provided (Social Interaction: cut-off score = 10; Communication: cut-off score = 8; Restricted and Repetitive Behaviors/Interests: cut-off score = 3), above which a child meets clinical criterion for ASD.

#### Global level of cognitive development

To measure global level of intelligence at follow-up, developmental age-appropriate instruments were used. The cognitive scale of the Bayley Scales of Infant and Toddler Development, 3rd edition (Bayley-3; [39]) was administered to 2–4 year old children. In the older children, four subtests of the Wechsler Preschool and Primary Scales of Intelligence, 3^rd^ edition (WPPSI-III; [40]) were used to estimate global level of intelligence (Block Design, Matrix Reasoning, Vocabulary, and Similarities). The WPPSI-III was selected, because there is a validated Dutch version. Total IQ estimates were calculated based on this short-form version of the WPPSI-III [41].

### Study procedures

**Fig. 1:**
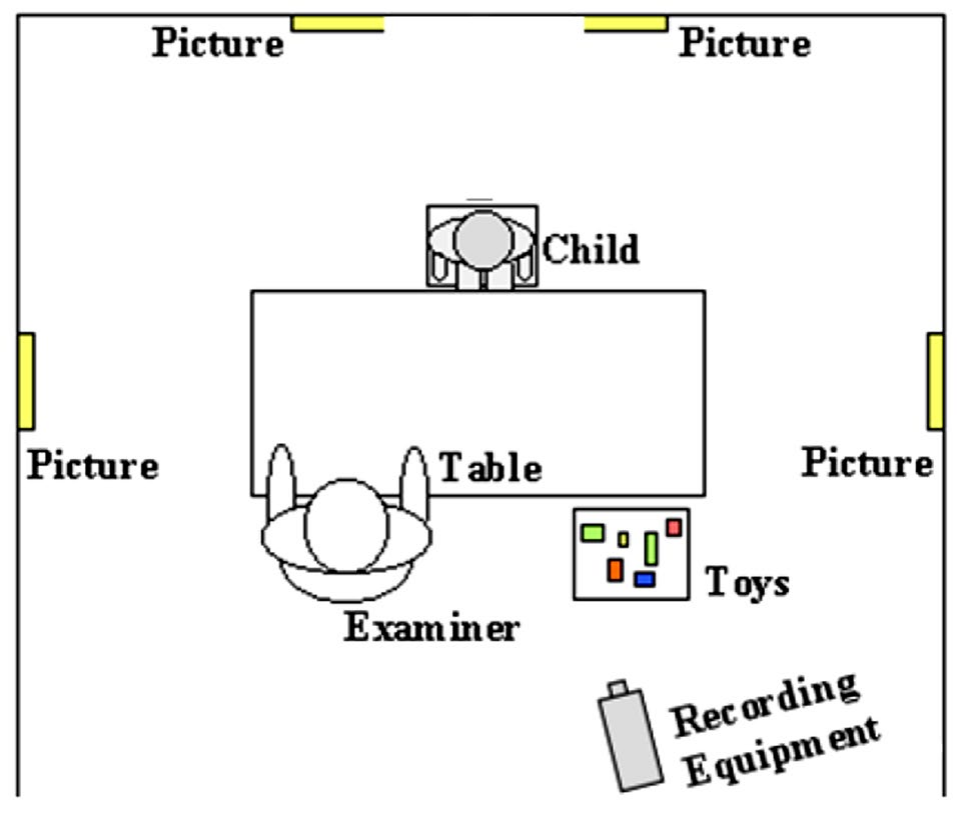
Set-up assessment room ESCS administration (adapted from: [32])

Assessment took place at various sites [Colorado (USA) and The Netherlands] either in a quiet room at the university or at home. To standardize the testing environment, the testing set-up and research protocols were identical at each research setting. Researchers from Leiden University were responsible for all project and data-management (i.e., training and supervision of researchers, processing and scoring of data). All assessments and questionnaires in the study were administrated by child psychologists in the Dutch or English language, depending on the first language of the child and parents. During the ESCS, the child was seated at a table across from a familiar examiner (see Fig. 1 for the set-up of the assessment room), and verbal interactions were kept to a minimum. The structured ESCS assessment was videotaped, with full-face view of the child and profile view of the experimenter. At a follow-up assessment, 1 year (= 12 months) after baseline, the Bayley-3, WPPSI-III, M-CHAT, and ADI-R were administered. The M-CHAT questionnaire was filled in by the primary caregiver of children aged 2–4 years, and the ADI-R interview was conducted with the primary caregiver of children with SCT, aged 4–8 year old children.

### Statistical analyses

Statistical Package for the Social Sciences (SPSS, version 25) was used for statistical analyses.

An independent sample *t* test was used to measure research site differences (The Netherlands vs. USA) on the outcome variables in the SCT group, and differences in ASD symptoms between two IQ groups (below average IQ; average IQ). Pearson’s *r* was used to assess correlations between age, global cognitive functioning, ASD symptoms, and Joint Attention. Hierarchical linear regression models were carried out to investigate the longitudinal relationships between Joint Attention and ASD symptoms, accounting for age effects (first step: age of the child; second step: Joint Attention). Differences on outcome measures between karyotype-subtypes and recruitment groups were measured with ANCOVAs, accounting for age effects. Statistical analyses were performed two-tailed, and statistical significance was set at *p* < 0.05 a priori. Effect sizes were calculated with *R*^2^ (explained variance).

## Results

### Comparison between research sites

First, to control for the potential impact of research site (and therefore for cultural bias/response tendencies) on outcomes of the study, the data of the two research sites were compared. No differences between research sites (The Netherlands, USA) were found for total scores on the primary measure of ASD symptoms, including the M-CHAT (*t* (26) =  − 1.20, *p* = 0.241), and for ADI-R total score (*t* (53) = − 1.53, *p* = 0.132). Therefore, data were collapsed across sites.

### Joint attention at baseline

The ESCS was successfully completed by 79 children (three children were not able to complete the task). Interrater reliability was measured based on a subsample of ten participants and shows an intraclass correlation coefficient (ICC) of 0.81–0.99 (for the Joint Attention scales collapsed together) which is considered to reflect excellent reliability [42]. Joint Attention on the baseline ESCS assessment was significantly correlated with age (*p* < 0.001). See Table 2 for descriptive statistics for Joint Attention in two SCT age groups (1–3 years; 3–7 years).

**Table 2:** Descriptive statistics of joint attention behaviors (initiating and responding) in children with sex chromosome trisomies

Joint attention (ESCS: raw score)	1–3 Year old *n* = 27		3–7 Year old *n* = 52		Correlations between JA and age
	min–max	*M* (SD)	min–max	*M* (SD)	Pearson’s *r*, *p* value
Initiating joint attention	2–43	18.07 (10.37)	0–89	30.00 (17.43)	*r* = .423, *p* < .001
Responding to joint attention (max. score = 12)	1–10	6.59 (3.08)	3–12	9.46 (1.58)	*r* = .523, *p* < .001

*JA* joint attention, *ESCS* early social communication scales

### Clinical risk for ASD at follow-up

The study group was divided into low risk and high risk based on the scoring algorithms of the M-CHAT and ADI-R as described above. To investigate overall clinical risk for the diagnosis of ASD in children with SCT across the 2–8 year age span, the M-CHAT and ADI-R data were collapsed. Out of 82 children with SCT, 18 children (22.0%) were classified as at clinical risk for ASD.

### ASD symptoms at different ages

To investigate symptoms of ASD at different developmental ages, the sample was divided into two age groups: children aged 2–4 years old (*N* = 28; 4 XXX, 20 XXY, 4 XYY), and children aged 4–8 years (*N* = 54; 20 XXX, 21 XXY, 13 XYY). The distribution of karyotypes (XXX, XXY, XYY) was different in the two age groups (*χ*^2^ (2) = 0.02, *p* = 0.018), due to the high number of XXY-participants in the younger age group.

#### 2–4 Year old children with SCT

ASD symptoms were assessed in the 2–4 years old (*N* = 28) using the M-CHAT. Within the SCT group, the mean score was 2.35 (*SD* = 2.06). Based on the manual of the M-CHAT, out of 28 children, 5 children were classified as moderate/high risk for ASD (17.9%), and 23 children (82.1%) were classified as low risk. Total score on the M-CHAT was not correlated with age (*r* = 0.137, *p* = 0.488).

#### 4–8 Year old children with SCT

The ADI-R interview was used to assess early ASD symptoms in the 4–8 year age group (*N* = 54). Within the SCT group, mean score in the Social Interaction + Communication domain was 20.22 (*SD* = 12.46), and in the Restricted Interests and Repetitive Behavior domain 2.57 (*SD* = 2.99). Within the SCT group, out of 54 children, 13 children (24.1%) scored above cut-off on all domains. For an overview of the percentages of children scoring above cut-off on the domains of ‘Social Interactions + Communication’, ‘Restricted Interests and Repetitive Behavior’, and on all domains, see Table 3. Total scores on the ADI-R were not correlated with age at follow-up (*r* = 0.209, *p* = 0.129).

**Table 3:** Percentage of children with SCT with ADI-R scores above cut-off

	% Children with SCT
Above cut-off on all domains	24.1%
Above cut-off on ‘Social Interactions’ + ‘Communication’	72.2%
Above cut-off on ‘Restricted Interests and Repetitive Behavior’	25.9%
Below cut-off on all domains	7.3%

*SCT* Sex Chromosome Trisomy

### Role of global cognitive functioning

Within the SCT group, ASD symptoms were not correlated with global cognitive functioning (M-CHAT total score and Bayley cognitive composite score: *r* = − 0.016, *p* = 0.938; ADI-R total score and WPPSI total IQ: *r* = − 0.298, *p* = 0.052). Because of the borderline *p *value of the negative correlation between ADI-R total scores and WPSSI total IQ (*p* = 0.052), we investigated whether ASD symptoms are more pronounced in children with SCT with a below average IQ. Total IQ was categorized into two groups (IQ < 84: below average; IQ > 85: average). The distribution of karyotypes (XXX, XXY, XYY) was similar between the two IQ groups (*χ*^2^ (2) = 0.37, *p* = 0.831). An independent *t* test was carried out to investigate differences in ASD symptoms between children with SCT in both IQ groups. No significant differences were found between children with SCT in the below average IQ group (*M* = 25.78, *SD* = 16.83), and the average IQ group (*M* = 21.25, *SD* = 14.70; *t* (43) = 0.81, *p* = 0.426). These results indicate that on average, SCT children with below average and average IQ have comparable amounts of ASD symptoms, although the correlation between lower cognitive scores and ASD symptoms in the older age group should be acknowledged.

### Predictive value of joint attention on ASD symptoms

To investigate whether initiating Joint Attention and responding to Joint Attention at baseline are predictors for early ASD symptoms at follow-up, hierarchical regression analyses were carried out in the two separate age groups, accounting for age effects.

#### 2–4 Year old children

No significant predictive relationship was found between initiating Joint Attention and ASD symptoms in children with SCT, aged 2–4 years (*F* (2,24) = 0.24, *p* = 0.789). However, responding to Joint Attention did significantly predict ASD symptoms in 2–4 years old children with SCT (*F* (2,24) = 4.24, *p* = 0.049; *b* = − 0.26, *β* = − 0.38, *R*^*2*^ = 0.15), indicating that a lower frequency of responses to Joint Attention significantly predict more ASD symptoms.

#### 4–8 Year old children

**Fig. 2:**
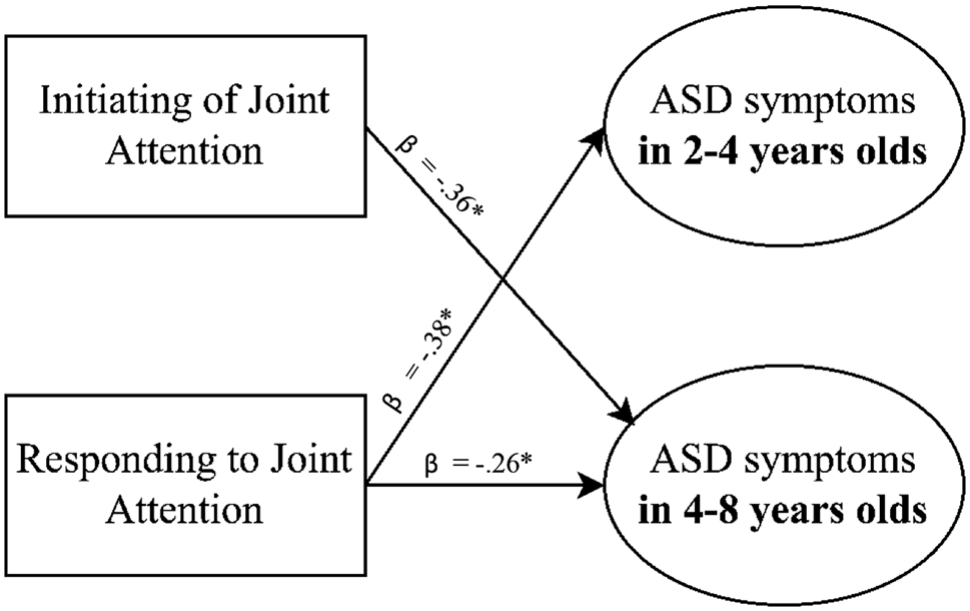
Significant longitudinal predictive relationships between Joint Attention and ASD symptoms in 2–4 year old children and 4–8 year old children with Sex Chromosome Trisomies. *p < .05

In 4–8 year old children with SCT, a significant predictive relationships was found between initiating Joint Attention and ASD symptoms (*F* (2,49) = 4.92, *p* = 0.011; *b* = − 0.30, *β* = − 0.36, *R*^*2*^ = 0.17). Similarly, a significant predictive relationship was found between responding to Joint Attention and ASD symptoms (*F* (2,49) = 3.23, *p* = 0.048; *b* = − 2.44, *β* = − 0.26, *R*^*2*^ = 0.12). These results indicate that lower frequencies of initiations of Joint Attention and responses to Joint Attention significantly predict more ASD symptoms in 4–8 year old children with SCT. See Fig. 2 for an overview of the significant relations between Joint Attention and ASD symptoms in the two age groups.

### Karyotype differences

As the distribution of karyotypes was different between the two age groups, ANCOVAs were carried out to measure differences between the various karyotypes on ASD symptoms in children aged 2–4 years (M-CHAT), and in children aged 4–8 years (ADI-R), accounting for age effects. For ASD symptoms in 2–4 year old children with SCT, no differences between karyotypes were found (*F* (1,24) = 0.53, *p* = 0.571). Similarly, for ASD symptoms in 4–8 year old children with SCT, no differences between karyotypes were found (*F* (1,51) = 0.88, *p* = 0.419), indicating a similar impact of the extra X or Y chromosome on ASD symptoms. See Table 4 for exact *M*, *SD*s and *p* values.

**Table 4:** Differences in ASD symptoms across karyotypes (M, SD)

ASD symptoms	XXX	XXY	XYY	*P* value
**2–4 Year old children**	*n* = 4	*n* = 20	*n* = 4	
M-CHAT: Total score	2.50 (1.73)	2.55 (2.28)	1.25 (0.50)	.571
**4–8 Year old children**	*n* = 20	*n* = 21	*n* = 14	
ADI-R: Total score	22.85 (13.41)	19.90 (12.78)	26.86 (17.26)	.419

*ASD* autism spectrum disorder

### Recruitment bias

Within the SCT group, we used ANOVAs to test for differences on total scores between the three recruitment groups (prospective follow-up after prenatal diagnosis/information seeking parents/clinically referred cases), accounting for age effects. There were no differences in recruitment groups on ASD symptoms in 2–4 year old children with SCT (*F* (1,24) = 0.24, *p* = 0.786) or in 4–8 year old children with SCT (*F* (1,51) = 0.53, *p* = 0.571). These results show that how children with SCT enrolled in the study is not related to their level of ASD symptoms. See Table 5 for exact *M*, *SD*s, and *p* values.

**Table 5:** Differences in ASD symptoms across recruitment strategies (M, SD)

ASD symptoms	Prospective follow-up	Information seeking parents	Clinically referred cases	*P* value
**2–4 Year old children**	*n* = 17	*n* = 9	*n* = 2	
M-CHAT: total score	2.59 (2.48)	2.11 (1.27)	1.50 (0.71)	.786
**4–8 Year old children**	*n* = 27	*n* = 13	*n* = 15	
ADI-R: total score	20.67 (14.67)	24.77 (13.80)	24.73 (14.28)	.523

*ASD* autism spectrum disorder, *SCT* sex chromosome trisomy

## Discussion

The current study aims to investigate the prospective impact of Sex Chromosome Trisomies (SCTs; XXX, XXY, XYY) on the appearance of early symptoms of Autism Spectrum Disorder (ASD), in children aged 1–8 years, with specific emphasis on the predictive value of Joint Attention on these ASD symptoms. The biological predisposition of SCTs allows us to study early social developmental pathways of a homogeneous group of children, and may be considered an ‘at risk’ group when it comes to neurobehavioral social adaptive development and related psychopathology [8, 17], since the risk for ASD in this group has been found to be about 15–30% in children and adolescents [8]. The results of our study add to the understanding of the early prospective contribution of the extra X or Y chromosome on clinical risk for behavioral defined neurodevelopmental disorder such as ASD, and of early Joint Attention mechanisms predicting these ASD symptoms. The results demonstrate that ASD symptoms are substantially higher in children with SCT between 2 and 8 years old as compared to the general population, with 22% of the children at clinical risk for ASD, based on parental reports.

The most important conclusion of our study is that clinical diagnostic levels of ASD symptoms in SCT may already be seen in 22% of very young children. Several studies examining risk for ASD symptoms at later ages in childhood and adolescence with various types of outcome measures and different SCT populations have found similarly elevated symptoms of ASD and related increased clinical diagnoses of ASD in individuals with SCT from school age into adolescence [5, 9, 10, 43]; see for a review: [8]. The results of the current study add to the literature by showing that a neurodevelopmental impact of the extra X or Y chromosome is already detectable in very young children with SCT that show ASD symptoms. ASD symptoms are similarly reported across all three karyotypes (XXX, XXY, and XYY). Ascertainment bias and site of recruitment were not found to be relevant to the percentages of ASD symptoms, indicating the robustness of these results.

By comparing two developmental age groups (2–4 years and 4–8 years), we were able to assess ASD symptoms during different developmental phases of early childhood. We found a relatively comparable impact of SCT on the appearance of early ASD symptoms at both age ranges (respectively, 18% and 24%). These ASD symptoms were not associated with global cognitive functioning scores, indicating that, on average, young children with SCT show an equal amount of ASD symptoms across different levels of cognitive functioning, although further examination of this relationship at older aged when cognitive assessments become more predictive of later functioning will be important.

Using the ADI-R interview, we assessed a profile of ASD symptoms in children from the age of 4 years and older. Although 24% of the children scores above cut-off on all domains of the ADI-R (Social Interaction + Communication, and Restricted Interests and Repetitive Behavior), we found that in 72% of the children scores on the Social Interaction + Communication domain reached clinical diagnostic thresholds. These results indicate a specific profile of ASD symptoms in young children with SCT, with relatively more vulnerabilities on the social and communicative domain, as compared to the whole conglomerate of ASD symptoms including restricted interests and repetitive behaviors.

Considering the impact of social impairments on interpersonal interaction and the development and maintenance of satisfying relationships with others [44], these early ASD symptoms may indicate an ‘at risk’ social pathway of a considerable percentage of children with SCT. However, it is important to note that there is also a subgroup of children with SCT that show no ASD symptoms in the clinical range. Thus, while a considerable percentage of parents express their concerns about the social development of their young child with SCT, there is also a group of young children with SCT with no or only some symptoms of ASD. Furthermore, it is important to emphasize that the presence of some ASD symptoms does not mean that the child meets full clinical criteria for a diagnosis of ASD.

The second aim of this study was to explore the predictive value of Joint Attention, as an early social-cognitive mechanism precursing ASD symptoms in young children with SCT. We used systematic behavior observations of Joint Attention during a structured play situation to evaluate the degree to which responding to another’s joint attention bids (i.e., Responding to Joint Attention) and the tendency to initiate joint attention episodes (i.e., Initiations of Joint Attention) are related to ASD symptoms. Our results demonstrate that in young children with SCT, the tendency to coordinate attention between social partners (i.e., Joint Attention) is predictive of ASD symptoms at 1-year follow-up. In SCT, the early development of Joint Attention abilities has proven to be an area of vulnerability [29], and the current results have implications for our understanding of the likely consequences of vulnerabilities in the capacity to form Joint Attention in young children with SCT. Low tendencies of following or initiating non-verbal communicative cues early in development may contribute to longer term impairments in adaptive social communication. Specifically, from earlier studies, it is known that Joint Attention is essential in social-cognitive development underlying social adaptive behavioral development; and the practice of Joint Attention provides experiences of perspective sharing for the child [22, 45]. A lower tendency to engage in Joint Attention therefore may lead to a cascade of negative developmental effects, not only in core social-cognitive skills, but also in language development and social adaptive behavioral functioning [46]; and indeed, we found that in SCT, Joint Attention mechanisms serve as early predictors of pathways for risk of severe social behavioral impairments, as indicated by symptoms of ASD.

Interestingly, we found differences in the two age groups with regard to the specific type of Joint Attention in predicting ASD symptoms at a specific age range: whereas only *responding* to Joint Attention predicts ASD symptoms in 2–4 year old children with SCT, both *responding* to and *initiating* Joint Attention predicted ASD symptoms in children aged 4 years and older. This finding supports the hypothesis that responding to Joint Attention and initiating Joint Attention have separate developmental trajectories and contribute differently to subsequent developmental outcomes [47, 48]. Reduced responses to Joint Attention are often observed during early childhood in children at risk for ASD. The initiation of Joint Attention seems to be even more critical and persistent in severe social impairments from early childhood into school age and adolescence [46]. Neuroimaging studies show that brain networks supporting *responding* to Joint Attention and *initiating* Joint Attention are partially distinctive (see for a review: Mundy [23]). Specifically, *initiating* Joint Attention is associated with increased activation of brain areas associated with reward processing [49, 50], suggesting that initiation of Joint Attention may be more related to social motivation, as compared to responding to Joint Attention that may be triggered more automatically and spontaneously. In conclusion, the results of the second part of our study indicate that, as children with SCT develop, challenges with the complex ability to initiate Joint Attention in which social motivation is believed to be involved, may be a pivotal precursor in the emergence of severe social impairments and the related risk for ASD symptoms; this, combined with the ability to spontaneously follow Joint Attention bids from social partners, is important across both age groups in this investigation.

The outcomes of our study could also provide insight in sex differences that are observed in neurodevelopmental disorders. ASD has been found to be more prevalent in males as compared to females and has different clinical manifestations in males and females [51, 52]. The understanding of these sex differences in ASD has important implications for tailored and individualized clinical care by guiding diagnosis and (preventive) intervention. The study of individuals with SCT provides a natural framework for the study of sex differences in neurodevelopmental disorders, as they provide us by a model for examining the effects of alterations in sex chromosome number on risk of ASD [53]. By comparing SCT conditions (XXX vs. XXY vs. XYY), we found no differences between karyotypes, indicating a similar impact of the extra X or Y chromosome on clinical risk for ASD between the age of 2–8 years. However, these findings are not in line with earlier studies that found increased ASD symptoms in school-aged boys and adolescents with XYY as compared to boys with XXY and typical controls (see for example: [5], and with increased ASD symptoms in 3–7 year old boys with XYY as compared to boys and girls with an extra X chromosome and typical controls [54]. Together, these data suggest that the Y chromosome in and of itself may be correlated with an increase in ASD susceptibility that become more pronounced in older age groups. However, it should be considered that the distribution of karyotypes (XXX, XXY, XYY) was unequal across age groups (2–4 years, 4–8 years). Although no effect of karyotype-subtype was found on ASD symptoms in both age groups in the current study, the specificity of early developmental pathways of karyotype-subtypes remain unclear, which calls for further studies with larger sample sizes. Direct comparisons between different SCT conditions in addition to comparison with age-related peers without an extra X or Y chromosome (XX, XY) would provide important evidence about the role of sex chromosomes on neurodevelopmental outcomes.

Overall, the results of the present study have important implications for clinical care. As ASD symptoms were found at the early age of 2 years, these findings underscore the importance of incorporating closely monitoring social adaptive functioning and screening for ASD symptoms in children with SCT as routine care within the first years of life. Because of the predictive relationship between Joint Attention and ASD symptoms as found in this study, difficulties with Joint Attention can serve as an early marker for an ‘at risk’ developmental profile and is a potential target for early intervention. Early detection and support of children ‘at risk’ is relevant for three reasons. First, children at risk for social impairments may have fewer social learning experiences compared to their peers, possibly leading to a cascade of negative developmental effects [22]. Second, parents of children with known SCT often experience stress and uncertainty about their child's development and their own parenting [55]. Early detection of developmental risk in children allows for appropriate support for parents by offering psychoeducation or coaching. Early support of parents might result in higher parent well-being and family adaptive functioning by means of improved positive psychological functioning and allow for learning effective coping strategies (see for examples about the efficacy of early parental support in the ASD literature: [56, 57]). Finally, preventive support and treatment early in life of children with SCT might reduce vulnerability for social impairments later in life. It is therefore of high relevance to evaluate the efficacy of neurocognitive and neurobehavioral interventions early in life of children with SCT. As early interventions are well established to support the early development of children with ASD and their families (see for a review: [58]), these early services may be beneficial for young children with SCT, as well [43]. Unfortunately, research evaluating the potential effects of early intervention and parental support in the SCT population remains scant, although there is recent promising evidence that early neurocognitive training can support social-cognitive development of 4–8 year old children with SCT [54, 59]*.* It is of urgent need to further investigate the possible efficacy and long-term outcomes of services and programs early in life of children with SCT, even more considering the need to offer supportive preventive interventions to the growing group of (prenatally) diagnosed children with SCT [3, 60].

The present study has several limitations, thus providing suggestions for future studies. First, our study on the early impact of SCT on ASD symptoms relies on parental reports which might represent bias. Behavioral symptoms of the child were not observed or discussed in a multidisciplinary team of clinicians to establish a shared clinical perspective. However, former studies demonstrated that parental assessment of ASD symptoms based on the M-CHAT and ADI-R correlates with behavioral observations of ASD symptomatology during clinical observations, for example during the Autism Diagnostic Observation Scale (ADOS; [33, 61]. Second, although a percentage of boys with XXY (Klinefelter Syndrome) received testosterone treatment (46%), the sample is not powered to study the efficacy of testosterone treatment on ASD symptoms. Randomized and placebo-controlled trials would provide more reliable insights into the effects of testosterone treatment in boys with Klinefelter syndrome (which is recently designed and running: PI Davis, NCT03325647). Finally, longer prospective follow-up of the cohort beyond 1 year is important to determine if these predictive relationships remain relevant as more complex social and communications skill develop, and whether Joint Attention and/or other factors are indeed predictive for a later clinical diagnosis of ASD.

To conclude, the current longitudinal study with a relatively large and international sample of young children with SCT demonstrates the appearance of early ASD symptoms in a subset of young children with SCT, from 2 years of age onward. Moreover, we found that Joint Attention, and especially the capacity to initiate Joint Attention, longitudinally predicts these ASD symptoms. These results advocate for close monitoring and early (preventive) support and intervention, aiming to optimize social adaptive development in young children with SCT.
